# Frailty in multiple sclerosis: A scoping review

**DOI:** 10.1016/j.msard.2024.106157

**Published:** 2024-11-05

**Authors:** Nida’ Al Worikat, Farzan Molaei, Anna Zanotto, Abbas Tabatabaei, Sharon G. Lynch, Bruce R. Troen, Jacob J. Sosnoff, Tobia Zanotto

**Affiliations:** a Department of Occupational Therapy Education, School of Health Professions, University of Kansas Medical Center, Kansas City, KS, United States; b Department of Occupational Therapy, School of Rehabilitation Science, The University of Jordan, Amman, Jordan; c Rehabilitation Research Center, Department of Physiotherapy, School of Rehabilitation Sciences, Iran University of Medical Sciences, Tehran, Iran; d Department of Physical Therapy, Rehabilitation Science, and Athletic Training, School of Health Professions, University of Kansas Medical Center, Kansas City, KS, United States; e Department of Neurology, School of Medicine, University of Kansas Medical Center, Kansas City, KS, United States; f Division of Geriatrics, Department of Internal Medicine, School of Medicine, University of Kansas Medical Center, Kansas City, KS, United States; g Landon Center on Aging, University of Kansas Medical Center, Kansas City, KS, United States; h Research Service, Kansas City Veterans Affairs Healthcare System, Kansas City, MO, United States; i Mobility Core, University of Kansas Center for Community Access, Rehabilitation Research, Education and Service, Kansas City, KS, United States

**Keywords:** Multiple sclerosis, Frailty, Aging, Review

## Abstract

This scoping review aims to provide a comprehensive overview of frailty in multiple sclerosis (MS), building upon the increasing number of studies that have recently begun to explore the potential impact of this age-related condition on the lives of people with MS (pwMS). A systematic search was conducted on PubMed, Scopus, Embase, Ovid MEDLINE, CINAHL, CENTRAL, Web of Science, PEDRO, and clinicaltrials.gov. The search results were limited to studies published between 2000 and 2024 without language restrictions. After the screening process, 11 studies (10 original articles and one conference paper) met the inclusion criteria and were included. The scope of the included studies was mainly aimed at quantifying frailty levels and prevalence in pwMS, investigating the association between MS clinical characteristics and frailty, and exploring the relationship between frailty and adverse clinical outcomes in pwMS. All studies concluded that frailty is highly prevalent in MS, with prevalences ranging from 17 % to 66 % (ambulatory patients only) and that pwMS have an early onset of frailty compared to the general non-MS population, as the mean age of the included participants ranged from 41.2 ± 9.0 to 58.6 ± 6.0 years. Higher disability levels, disease duration, and progressive MS subtypes were commonly associated with frailty. In addition, several studies showed that frailty was strongly associated with reduced walking performance, lower sleep quality, fatigue, lower quality of life, falls, primary care visits, and mortality. In conclusion, frailty represents a common yet underinvestigated clinical concern for the MS community.

## 1. Introduction

Multiple sclerosis (MS) is a chronic inflammatory autoimmune disorder affecting the central nervous system and represents the most prevalent non-traumatic disabling condition impacting young adults (Dobson and Giovannoni, 2019). Nearly three million people live with this condition worldwide (Walton et al., 2020). Typically, MS is diagnosed in individuals between the ages of 20 and 30 and is characterized by sensory, motor, and cognitive impairments, as well as fatigue and depressive symptoms (Benedict et al., 2020; Bethoux, 2013; Jørgensen et al., 2017; Manjaly et al., 2019; Timkova et al., 2021). These symptoms can significantly impact several aspects of daily functioning, leading to a significant decline in the individual’s overall quality of life (Fidao et al., 2021; McGinley et al., 2021). In addition, people with MS (pwMS) have a higher risk of falls, utilization of healthcare facilities, and younger age at death compared to the general non-MS population (Marrie et al., 2015a; Nilsagard et al., 2015). Importantly, the prevalence and incidence of MS in the U.S. have been increasing steadily over the last decade (Hittle et al., 2023). Likewise, the median age of the U.S. MS population has also increased (Vaughn et al., 2019). Consequently, the aging of pwMS will inevitably pose new challenges for their healthcare and the MS community (Graves et al., 2023). One of these challenges will be to ensure that age-related syndromes, such as frailty, are timely recognized and effectively managed in pwMS.

Frailty is a biological syndrome of decreased reserve and resistance to stressors arising from cumulative declines across multiple physiologic systems and causing vulnerability to adverse outcomes (Rockwood and Howlett, 2018). Several ways of evaluating frailty exist. However, the two main conceptualizations of frailty are physical frailty, as typically assessed by the Fried phenotype (Fried et al., 2001), and the deficit accumulation model, as assessed through the frailty index (Rockwood and Mitnitski, 2007). The Fried phenotype is the most commonly used measure of frailty and defines five core components of frailty: slowness (slow gait speed), weakness (low handgrip strength), inactivity (low self-reported physical activity), exhaustion (self-reported fatigue), and shrinkage (self-reported unintentional weight loss). Individuals who meet at least three of these five components are defined as frail (Fried et al., 2001). The strengths of this operationalization of frailty are its cost-and time-effectiveness, as well as its large evidence base in terms of predicting negative health outcomes in various clinical populations (Dent et al., 2016). On the other hand, the deficit accumulation model quantifies frailty on a continuum based on the presence or absence of several health-related deficits (usually 30–40 deficit items are required) that are used to calculate a frailty index (Rockwood and Mitnitski, 2007; Searle et al., 2008). Importantly, the health-related deficits used to calculate the frailty index encompass various domains of function (e.g., physical, cognitive, psychological, and social function), making the deficit accumulation model a more comprehensive conceptualization of frailty.

Given that MS impacts several physiological systems and is associated with several comorbidities (Marrie et al., 2015b), it is not surprising that pwMS are often frail. Although frailty has traditionally been studied in the context of aging populations, this biological syndrome can occur throughout the lifespan, especially in individuals with chronic conditions such as MS (Zazzara et al., 2019). Notably, a recent population-based study conducted by Hanlon et al. (2018) concluded that pwMS had a 15-times higher risk of being frail compared to age-matched individuals without MS, making MS the top long-term disorder associated with frailty among the chronic health conditions examined in their study. There is also compelling evidence that pwMS become frail at a younger age compared to individuals without MS (Ayrignac et al., 2021; Belvisi et al., 2021). For instance, several recent studies have reported that approximately one-to two-thirds of pwMS meet objective diagnostic criteria for frailty (Ayrignac et al., 2021; Frau et al., 2023; Kumar et al., 2024; Zanotto et al., 2022a). In addition, frailty within MS is strongly associated with common MS-related symptoms and adverse clinical outcomes, such as falls, independent of neurological disability (Zanotto et al., 2022a; Zanotto et al., 2023). This raises the possibility that frailty and disability are related yet independent of each other in pwMS. These observations suggest that frailty should be a common clinical concern in MS.

Although frailty in MS has garnered considerable attention over the last five years, the impact of this syndrome on the lives of pwMS remains an under-investigated area of research. To the best of our knowledge, there are no published review articles synthesizing the current knowledge base on frailty in pwMS. Therefore, this scoping review aims to comprehensively examine the existing evidence on frailty in the context of MS and systematically explore the literature’s depth, scope, and nature to identify research gaps and inform future research directions.

## 2. Methods

### 2.1. Data sources and search strategy

We performed a scoping review following the PRISMA-ScR reporting guidelines (Tricco et al., 2018). A systematic exploration of the literature was conducted across a range of databases, including PubMed, Scopus, Embase, Ovid MEDLINE, CINAHL, CENTRAL, Web of Science, PEDRO, and clinicaltrials.gov (all database strategies are reported in Appendix A). We focused on all studies published from 2000 to March 2024 without setting any language restrictions. The rationale for conducting the database searches starting from the year 2000 was based on the fact that the landmark paper by Fried et al. (2001) providing the first widespread operationalization of frailty was published in 2001. The search terms utilized covered a broad spectrum, including “multiple sclerosis,” “demyelinating disease,” “autoimmune neurological disorder,” “central nervous system demyelination,” “neuroinflammatory disease,” “frailty,” “frail elderly,” “frail,” “sarcopenia,” and “sarcop,” as fully detailed in Appendix A.

### 2.2. Selection criteria for studies

The inclusion criteria were restricted to original studies in the following categories: 1) clinical trials, 2) case reports, 3) observational studies, or 4) conference papers. Only studies that expressly investigated frailty in pwMS were considered for inclusion. More specifically, studies had to incorporate a formal assessment of frailty, such as the Fried frailty phenotype, frailty index, or other internationally recognized frailty assessment tools, to be eligible for inclusion. Lastly, we exclusively considered studies involving adult human participants with a confirmed diagnosis of MS. For exclusion criteria, review or meta-analysis, book chapters, or protocol studies without results were excluded. Additionally, editorials or letters to the editor were not considered for inclusion.

### 2.3. Study selection and data extraction

Two authors (NA and FM) evaluated the studies’ eligibility for inclusion in the review by checking the titles, abstracts, keywords, and, subsequently, the full text. Each investigator examined all the articles separately and then documented the findings individually using a consistent form. The authors convened together to identify any disparities between their findings. In cases of disagreement, a third author (TZ) was consulted to find a resolution.

Using a customized form, two authors (NA and FM) independently extracted information relevant to the purpose of this scoping review. This included aspects such as study design, country where the study was conducted, sample size, characteristics of the study population (i.e., age, sex, type of MS), and study objectives. Concerning frailty, the following information was extracted: the conceptualization of frailty (e.g., physical frailty, deficit accumulation model, etc.), the operationalization of frailty (i.e., the specific tool or measure that was used to assess frailty), frailty prevalence, and lastly, the main study findings. Regular meetings were held among the authors to discuss their findings and ensure the continual refinement of the data-charting form in accordance with the emerging results.

## 3. Results

### 3.1. Study selection

**Fig. 1.**
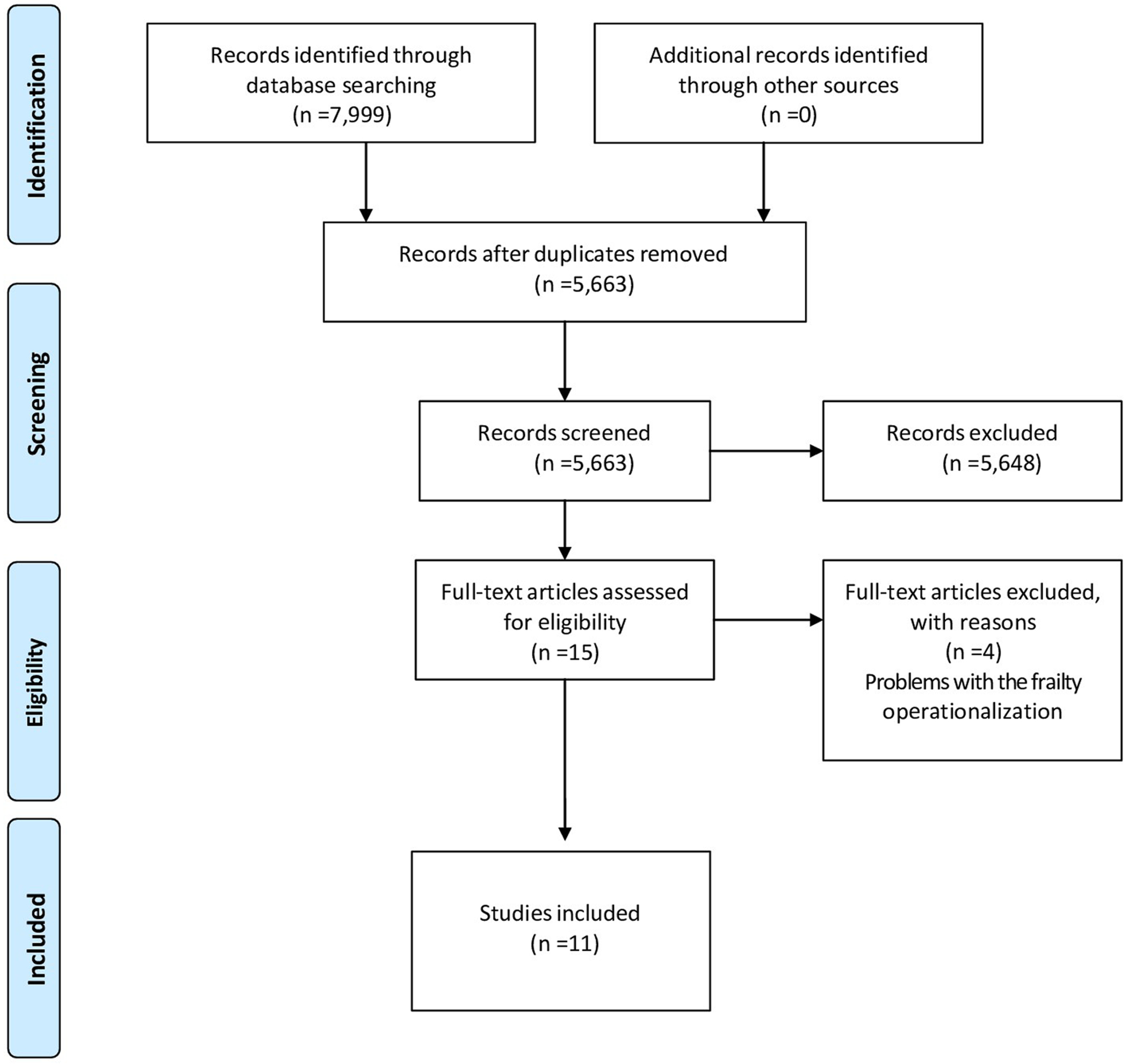
PRISMA Flow diagram illustrating the literature search and article screening.

Through the systematic web-based search, we identified 7999 articles. Of these, 2336 were discarded due to duplication. Among the remaining 5663 studies, 5648 were deemed irrelevant based on the titles’ and abstracts’ screening results. Consequently, 15 studies underwent full-text assessment. Following a thorough evaluation, four studies were further excluded, leaving us with 11 studies included in the present scoping review (Fig. 1). Ten studies were original articles (Ayrignac et al., 2021; Baione et al., 2023; Belvisi et al., 2021; Frau et al., 2023; Hanlon et al., 2018; Pradeep Kumar et al., 2024; Tartaglia et al., 2023; Zanotto et al., 2022a, b; Zanotto et al., 2023), while one (Palladino et al., 2022) was a conference abstract.

### 3.2. Study characteristics

Table 1 summarizes the characteristics of the 11 studies included in the review. In terms of study design, all the included studies were observational. Two studies (18.2 %) had a prospective study design (Baione et al., 2023; Hanlon et al., 2018), while the remaining nine investigations (81.8 %) had a retrospective cross-sectional study design (Ayrignac et al., 2021; Belvisi et al., 2021; Frau et al., 2023; Palladino et al., 2022; Pradeep Kumar et al., 2024; Tartaglia et al., 2023; Zanotto et al., 2022a, b; Zanotto et al., 2023). The studies encompassed 15,652 participants diagnosed with MS. Sample sizes ranged from 19 to 12,251, and the mean age of participants ranged between 41.2 ± 9.0 years and 58.6 ± 6 years. Women comprised the majority (70.92 %) of the participants. Ten studies focused on pwMS as the primary population, whereas one study included pwMS within a broader population of 493, 737 middle-aged and older adults (Hanlon et al., 2018). Most studies encompassed all three subtypes of MS, namely relapsing-remitting MS (RRMS), primary progressive MS (PPMS), and secondary progressive MS (SPMS). The 11 studies were conducted in the United States (*n* =4), Italy (*n* = 4), the United Kingdom (*n* = 2), and Canada (*n* = 1).

### 3.3. Study findings

The objectives of the included studies mainly encompassed quantifying frailty levels (and/or frailty prevalence) in pwMS, understanding what clinical characteristics are associated with frailty in MS, and investigating the relationship between frailty and common MS-related symptoms or adverse clinical outcomes in pwMS (Table 2). The deficit accumulation model was predominant among the conceptual frameworks employed, with nine studies adopting it (Ayrignac et al., 2021; Baione et al., 2023; Belvisi et al., 2021; Palladino et al., 2022; Pradeep Kumar et al., 2024; Tartaglia et al., 2023; Zanotto et al., 2022a, b; Zanotto et al., 2023). Two studies used the physical frailty model (Hanlon et al., 2018; Ayrignac et al., 2021), and one used another multidimensional conceptualization of frailty (Tilburg frailty indicator; Frau et al., 2023(. The studies that used the deficit accumulation model operationalized frailty through frailty indices based on 30 to 50 health-related deficit items. One study used the electronic Frailty Index (Palladino et al., 2022). On the other hand, the two studies evaluating physical frailty used the Fried frailty phenotype or adaptations of it. The mean frailty index from the nine studies using the deficit accumulation model ranged between 0.13 ± 0.10 and 0.54 ± 0.13. The prevalence of frailty emerging from the two studies that used the Fried frailty phenotype was 17 % and 28 %, respectively.

Only two studies compared frailty between pwMS and age-matched individuals without MS and found that frailty was significantly higher in pwMS than their non-MS counterparts (Ayrignac et al., 2021; Hanlon et al., 2018). Several demographic and clinical characteristics, such as age, female sex, education levels, disability and comorbidity levels, disease duration, and progressive MS, were found to be associated with frailty (Table 2). The three most common conditions associated with frailty were disability, disease duration, and progressive MS. Among these, higher disability was the most commonly reported. Indeed, six studies examined the association between disability levels, evaluated through the Expanded Disability Status Scale (EDSS), and various frailty measures (Ayrignac et al., 2021; Baione et al., 2023; Belvisi et al., 2021; Frau et al., 2023; Tartaglia et al., 2023; Zanotto et al., 2022a). All studies found significant correlations between higher EDSS and frailty measures, with correlation coefficients ranging from 0.37 (Baione et al., 2023; Zanotto et al., 2022a) to 0.62 (Ayrignac et al., 2021). In addition, frailty was associated with lower autonomy and quality of life and adverse clinical outcomes, such as falls, higher number of primary care visits, and mortality. Moreover, frailty was associated with a number of MS-related symptoms, encompassing worse walking performance, fatigue, and sleep problems.

## 4. Discussion

The investigation of frailty in pwMS has the potential to offer valuable insights into the complex interplay between MS and age-related health vulnerabilities. The progressive aging of MS populations worldwide underscores the critical need to identify strategies to minimize the adverse effects of age-related conditions on the health of pwMS (Graves et al., 2023). This scoping review aimed to provide a comprehensive overview of the existing literature on frailty in MS, highlighting its prevalence, associated clinical characteristics, and relationship with adverse clinical outcomes. The findings showed that frailty is highly prevalent among pwMS compared to the general population and individuals living with other long-term conditions. Higher disability levels, disease duration, and progressive MS subtypes were frequently associated with frailty measures across the included studies. In addition, frailty was associated with a range of signs and symptoms, such as reduced walking performance, fatigue, and lower sleep quality, as well as adverse health outcomes such as falls, primary care visits, and mortality in pwMS.

### 4.1. Prevalence of frailty in MS

Owing to the high heterogeneity in frailty operationalizations used across the various studies, conducting any meta-prevalence analyses of frailty in pwMS is not possible. Most studies (*n* = 9, 81.8 %) used the deficit accumulation model (i.e., frailty index approach) to conceptualize frailty. An advantage of this model is that it considers frailty as a multidimensional syndrome resulting from deficits across multiple health domains, including, but not limited to, physical, psychological, and social aspects (Rockwood and Mitnitski, 2007). Since MS is known to affect all of these health-related domains, the deficit accumulation model may be a valuable tool in assessing MS-related frailty. Another advantage of the deficit accumulation model is that it allows researchers to generate a customized frailty index using different health-related items from pre-existing datasets, as long as some guiding principles are followed (Searle et al., 2008). This also likely explains why the deficit accumulation model was common across the included studies. Although the customizability of the frailty index approach represents a valued feature, it also has several drawbacks. One of these is that it is not possible to compare frailty indices that were computed using different health-related variables (Searle et al., 2008), which explains the discrepancies in frailty prevalence estimates observed in the included studies. Particularly, despite using similar cut-off values (Clegg et al., 2016), frailty indices that predominantly included comorbidities and biomarker values resulted in lower frailty prevalences (e.g., 3.8 %; Palladino et al., 2022), while frailty indices encompassing a relatively high number of ordinally expressed deficit items resulted in higher frailty prevalences (e.g., 66 %; Zanotto et al., 2022a). On the other hand, the two studies that assessed physical frailty using adaptations of the Fried phenotype resulted in more aligned frailty prevalence estimates (i. e., 17 % and 28 %; Ayrignac et al., 2021; Hanlon et al., 2018). Notably, the different estimates of frailty prevalence were also influenced by the clinical characteristics of participants in the individual studies. For instance, the prevalence of frailty in non-ambulatory pwMS (upwards of 90 %) was much higher than in individuals with mild-to-moderate disability (Zanotto et al., 2022b). Regardless of the operational definitions of frailty, findings from this review strongly suggest that frailty is highly prevalent among pwMS. Most of the included studies were not sampled in such a way as to measure the prevalence of frailty. Indeed, several studies included small convenience samples or used specific recruitment strategies that would influence the prevalence of frailty. Thus, a very broad range of frailty prevalence was reported, suggesting that the true prevalence of frailty in MS is still unknown. Importantly, studies conducted in prevalent ambulatory pwMS reported that between 17 % and 66 % of participants met objective diagnostic criteria for frailty. For comparison purposes, the geriatric literature suggests that 7 % to 15 % of older adults (i.e., people aged ≥65) are frail (Clegg et al., 2016; Fried et al., 2001). Mirroring these data, 8 % of the non-MS control group from Ayrignac et al. (2021) (mean age: 61.0 ± 6.5 years) were classified as frail, as opposed to 28 % of pwMS. A second important finding was that frailty appears to develop earlier in MS, as the mean age of pwMS in the reviewed studies ranged from 41.2 ± 9.0 to 58.6 ± 6.0 years.

### 4.2. Clinical characteristics associated with frailty in MS

The three most common clinical characteristics associated with frailty in pwMS were higher disability, longer disease duration, and progressive MS subtypes (PPMS and SPMS; Ayrignac et al., 2021; Belvisi et al., 2021; Frau et al., 2023; Marrie et al., 2015b; Zanotto et al., 2022a; Zazzara et al., 2019). These findings underscore the substantial overlap between disability and frailty, which is also frequently observed among non-MS geriatric populations (Theou et al., 2012). Specifically, individuals with PPMS are also known to have higher disability levels than people with RRMS (Thompson, 2004). Analogously, individuals with SPMS have both a longer disease duration and a higher disability than people with RRMS (Tartaglia et al., 2023). Therefore, the relationship between longer disease duration, progressive MS subtypes, and frailty in pwMS may reflect the association between the accumulation of MS-related disability and frailty levels. Interestingly, this observation raises the question of whether frailty and disability represent the same phenomenon or whether they are conceptually distinct in the context of MS. Several findings emerging from this scoping review suggest that, while there is a degree of overlap, frailty, and disability are independent of each other in pwMS. For instance, two studies reported that frailty was associated with falls independently of neurological disability, as assessed by EDSS and PDDS (Zanotto et al., 2022a, b). Moreover, in the study by Zanotto et al. (2022b), a range of moderate to severe frailty was found within a homologous disability category (PDDS = 7.0). In another study, the EDSS and the frailty index were differently associated with walking quality and quantity measures, as assessed by wearable sensors (Zanotto et al., 2023). Particularly, the EDSS was more strongly associated with measures of walking quality, while the frailty index fully mediated the relationship between the EDSS and global physical activity, suggesting that frailty rather than disability may be primarily responsible for the total amount of physical activity performed by pwMS. In addition, a recent study by Frau et al. (2023) reported a significant association between the physical component of the Tilburg frailty indicators and the EDSS. However, frailty’s psychological and social components were not correlated with the EDSS. This observation may reflect the notion that the “resilience” component of frailty, also known as “resistance to stressors”, is influenced not only by physiological reserves but also by psychological and social factors (Hoogendijk et al., 2019). This is also likely responsible for the critical conceptual difference between frailty and disability in the MS population.

The relationship between sex and frailty in the context of MS is of interest due to the well-documented sex imbalance in pwMS (~75 % of pwMS are female; Hunter, 2016). The geriatric literature indicates that older women tend to have a higher prevalence of frailty than older men, which can be attributed to women’s higher life expectancy and various biological, social, environmental, lifestyle, and nutritional factors (Gordon and Hubbard, 2020; McGuigan et al., 2017). Mirroring these findings, Belvisi et al. (2021) reported higher frailty index scores in women with MS compared to men with MS in an age-matched comparison. Similarly, Palladino et al. (2022) found that frailty and depression were more strongly associated with mortality in women than in men with MS. Therefore, it is plausible that the higher prevalence of MS among women may contribute to the overall higher proportion of frailty, as well as its clinical relevance, in the MS population.

### 4.3. Frailty, MS-related signs and symptoms, and adverse outcomes

Several studies provided insights on the potential clinical implications of frailty in MS by investigating the relationship between frailty measures and common MS-related signs and symptoms or adverse clinical outcomes in pwMS. An important caveat of these studies is that, due to their observational designs, the level of evidence concerning the role played by frailty in aggravating common MS-related problems remains scarce. However, several findings are noteworthy in this early stage of research. For example, cross-sectional studies showed that frailty is strongly associated with reduced free-living walking performance (Zanotto et al., 2023), lower sleep quality (Pradeep Kumar et al., 2024), fatigue (Belvisi et al., 2021), and lower quality of life (Frau et al., 2023) in pwMS. Relatedly, frailty within MS was also strongly and independently (of age, sex, and disability) associated with a history of falls (Zanotto et al., 2022a, b). Accidental falls are one of the main clinical concerns in pwMS (Cameron and Nilsagard, 2018). In two independent studies, it was found that frailty indices were more strongly associated with falls than the EDSS and PDDS, raising the possibility that frailty assessment tools may be better suited to predict falls than common neurological disability assessments in the MS community (Zanotto et al., 2022a, b). Notably, a recent study by Palladino et al. (2022) provided evidence that frailty is also associated with other adverse clinical events, such as a higher number of primary care visits and mortality in pwMS. Overall, the sum of these observations indicates that frailty is a common clinical concern for the MS community, and there is a critical need to identify strategies to counteract frailty in pwMS.

### 4.4. Limitations and research gaps

The relatively low number of included studies (*n* = 11) represents the main limitation of this scoping review. Nevertheless, 10 out of 11 studies were published in the last four years, which underlines the increasing interest in this area of research. Most studies were cross-sectional, which limits any inference on the presumed causality between frailty and MS-related problems. In this respect, more studies with a prospective design would be needed to investigate the association between frailty and adverse clinical outcomes. Moreover, prospective studies would allow researchers to understand the frailty trajectory over time in pwMS. In addition, no study has yet attempted to investigate the effects of pharmacological or non-pharmacological interventions as a strategy to reduce frailty in pwMS. To date, there is only one published study protocol for an ongoing pilot randomized controlled trial looking at the feasibility of multimodal exercise to minimize frailty in frail pwMS (Zanotto et al., 2024). Therefore, there is a critical need to study whether frailty is modifiable in the MS community.

In addition, the observation that pwMS have an early onset of frailty raises the possibility that either the etiology of frailty in MS may differ from age-related frailty or that MS may represent a model of accelerated aging. From this perspective, there is currently a mechanistic research gap in the literature, and further research into the biology of frailty in pwMS is warranted to understand the pathophysiology of MS-related frailty better. Last but not least, the predominance of studies from Western countries highlights a notable geographical limitation, potentially limiting the generalizability of the findings to diverse MS populations with distinct sociodemographic and healthcare contexts. Therefore, further research is needed to understand the epidemiology of frailty in MS more comprehensively across different cultural and geographical regions.

## 5. Conclusions

This scoping review is the first to provide a comprehensive overview of frailty in MS. The available literature strongly suggests that frailty is highly prevalent in MS and that pwMS have an earlier onset of frailty compared to the general non-MS population. More specifically, frailty seems to be more common among middle-aged pwMS compared to older adults without MS. Frailty within MS was commonly associated with higher disability, disease duration, and progressive MS subtypes. Although frailty and disability present substantial overlap, evidence suggests they are conceptually distinct in pwMS. In addition, several studies reported that frailty is strongly associated with common MS-related signs and symptoms, such as walking and sleeping problems, fatigue, lower quality of life, falls, primary care visits, and mortality in pwMS. Therefore, frailty may represent an underinvestigated clinical realm in the MS community. Future research focusing on elucidating the underlying biological factors of frailty in MS is warranted. Finally, this scoping review highlights an emerging need to identify strategies to manage and counteract frailty to maximize the health of pwMS.

## Supplementary Material

Supplement

Supplementary material associated with this article can be found, in the online version, at doi:10.1016/j.msard.2024.106157.

## Figures and Tables

**Table 1:** Characteristics of the included studies.

Study	Title	Country	Study design	Sample size	Population	Age	Sex
Ayrignac et al., 2021	Frailty in ageing persons with multiple sclerosis	Canada	Cross-sectional study	• People with MS *n* = 80 • Control group *n* = 37	• People with MS [RRMS *n* = 56 (70 %), SPMS *n* = 17 (21 %), PPMS *n* = 7 (9 %)] • People without MS	• PwMS mean ± SD: 58.6 ± 6.0 years • Control group mean ± SD: 61.0 ± 6.5 years	• Male (30.0 % pwMS; 35.0 % control) • Female (70.0 % pwMS; 65.0 % control)
Baione et al., 2023	Frailty and relapse activity in multiple sclerosis: A longitudinal observation	Italy	Longitudinal observation study	471	People with MS [RRMS 85.5 %, SPMS 12.2 %, PPMS 2.3 %]	Mean ± SD: 43.7 ± 11.4 years	• Male (33.5 %) • Female (66.5)
Belvisi et al., 2021	Operationalization of a frailty index in patients with multiple sclerosis: A cross-sectional investigation	Italy	Cross-sectional study	745	People with MS [RRMS 83.3 %, SPMS 14.2 %, PPMS 2.5 %]	Mean ± SD: 48.2 ± 11.7 years	• Male (32.0 %) • Female (68.0 %)
Frau et al., 2023	Multidimensional frailty and its association with quality of life and disability: A cross-sectional study in people with multiple sclerosis	Italy	Cross-sectional study	208	People with MS [PPMS *n* = 2 (1 %), RRMS *n* = 186 (89.4 %), SPMS *n* = 20 (9.6 %)]	Mean ± SD: 44.0 ± 11.0 years	• Male (25.0 %) • Female (75.0 %)
Tartaglia et al., 2023	Neurophysiological and clinical biomarkers of secondary progressive multiple sclerosis: A cross-sectional study	Italy	Cross-sectional study	19	People with MS [RRMS *n* = 13 (68.4 %), SPMS *n* = 6 (31.6)]	RRMS, mean ± SD: 41.2 ± 9.0 years SPMS, mean ± SD: 53.3 ± 5.2 years	• Male (36.8 %) • Female (63.2 %)
Zanotto et al., 2022b, a	Frailty among people with multiple sclerosis who are wheelchair users	USA	Cross-sectional study	45	People with MS [RRMS *n* = 18 (40 %), PPMS *n* = 9 (20 %), SPMS *n* = 16 (35.6 %), unclear MS type *n* = 2 (4.4 %)]	Median [IQR]: 60.0 [16.0] years	• Male (17.8 %) • Female (82.2 %)
Hanlon et al., 2018	Frailty and pre-frailty in middle-aged and older adults and its association with multimorbidity and mortality: a prospective analysis of 493 737 UK Biobank participants	UK	Prospective, population-based cohort study	All participants *n* = 493,737; People with MS *n* = 1540	Middle-aged and older adults	Age range: 37–73 years	• Male (45.6) • Female (54.4 %)
Zanotto et al., 2022a, b	Frailty and Falls in People Living With Multiple Sclerosis	USA and Israel	Cross-sectional study	118	People with RRMS	Mean ± SD: 48.9 ± 10.0 years	• Male (25.4 %) • Female (74.6 %)
Zanotto et al., 2023	Association Between Frailty and Free-Living Walking Performance in People With Multiple Sclerosis	USA and Israel	Cross-sectional study	99	People with RRMS	Mean ± SD: 49.3 ± 9.8 years	• Male (26.3 %) • Female (73.7 %)
Kumar et al., 2024	Association between frailty and sleep quality in people living with multiple sclerosis and obesity: An observational cross-sectional study	USA	Cross-sectional study	76	People with obesity and MS [RRMS *n* = 71 (93.42 %), SPMS *n* = 2 (2.63 %)]	Mean ± SD: 47.6 ± 10.9 years	• Male (18.4 %) • Female (81.6 %)
Palladino et al., 2022*	The interface of frailty, depression, vascular disease, and mortality in over 12,000 people with Multiple Sclerosis in England: a population-based retrospective cohort study	UK	Population-based retrospective cohort study	12,251	People with MS	N/A	N/A

*Notes:* SD: standard deviation; IQR: Interquartile range; RRMS: Relapsing-Remitting MS; SPMS: Secondary Progressive MS; PPMS: Primary Progressive MS;* Conference abstract.

**Table 2:** Main study findings.

Study	Study aims	Conceptualization	Operationalization	Frailty levels/prevalence	Main findings
Ayrignac et al., 2021	To evaluate the prevalence of frailty in pwMS compared to controls over 50 years and determine the primary characteristics associated with frailty in pwMS.	Physical frailty and deficit accumulation model	• Fried frailty phenotype (FP) • Frailty Index (50 variables)	• FI mean ± SD: 0.21 ± 0.12 (in pwMS), 0.11 ± 0.08 (in controls) Frailty prevalence (FP): 28 % (in pwMS), 8 % (in controls)	• Frailty was significantly higher in pwMS compared to controls in the two measures, FP and FI (*p* = 0.017 and *p <* 0.0001), respectively. • There was a significant association between FI and comorbidities, education level, and disease duration. • PwMS with EDSS ≥ 3 were more frequently frail and had a higher FI than those with EDSS < 3. Additionally, pwMS with EDSS < 3 exhibited a higher FI than controls. • A significant correlation between FI and EDSS was observed in pwMS *(p* < 0.0001).
Baione et al., 2023	To explore the clinical association between frailty and disease activity among outpatients with MS receiving care at tertiary services.	Deficit accumulation model	Frailty Index (42 variables)	• FI mean ± SD: 0.13 ± 0.11	FI score negatively correlated with the incidence of relapses. • FI values were significantly higher in SPMS compared to RRMS patients (*p* < 0.000001). • Significant correlations between FI and age, disease duration, and EDSS score were detected (*p* < 0.000001). • In patients who experience relapses, a significant association was found between FI and relapse-associated worsening (*p* = 0.006).
Belvisi et al., 2021	To explore the association between frailty and the clinical manifestations of MS.	Deficit accumulation model	Frailty Index (42 variables)	• FI mean ± SD: 0.13 ± 0.10 • Frailty prevalence: 17.2 %	• FI scores positively correlated (*p* < 0.001) with fatigue, age, MS disease duration, the number of prior DMTs, and present symptomatic treatments. • Patients with progressive MS (PPMS & SPMS) had higher FI median scores compared to RRMS. • FI scores were higher among female participants than males with the same age range. • A positive correlation was found between FI scores and EDSS scores (*p* < 0.001). • Male sex (*p* = 0.03), EDSS scores (*p* < 0.001), and FI scores *(p* = 0.02) were significantly associated with SPMS.
Frau et al., 2023	To assess frailty using the Tilburg frailty indicator (TFI) and to investigate its relationship with autonomy, quality of life, and disability.	Multidimensional frailty (physical, psychological, and social)	Tilburg frailty indicator (TFI)	• Total TFI score mean ± SD: 5.7 ± 3.0 • Frailty prevalence: 62.5 %	• EDSS score was associated with both the overall and physical frailty scores (*p* < 0.001); PwMS with higher EDSS scores were more likely to be frail. • Frail pwMS exhibited greater autonomy impairment *(p* = 0.017) and poorer quality of life (*p* < 0.001). No significant associations were observed between the EDSS score and the psychological and social frailty domains.
Tartaglia et al., 2023	To examine the association between the neurophysiological index and the frailty index in pwMS.	Deficit accumulation model	• Frailty Index (42 variables)	• FI mean ± SD: 0.14 ± 0.10 (RRMS), 0.27 ± 0.09 (SPMS)	• The FI scores were significantly higher in patients with SPMS (0.27±0.09) than those with RRMS (0.14 ± 0.10) *(p* = 0.02). • There was a significant difference between the neurophysiological index scores in RRMS (0.19±0.19) and SPMS (0.92 ± 0.07) *(p <* 0.001). • FI and neurophysiological index were positively associated with each other and with the EDSS.
Zanotto et al., 2022b, a	To assess frailty via the deficit accumulation model and to investigate the relationship of frailty with MS clinical subtypes, disease duration, and fall history among wheelchair users with MS.	Deficit accumulation model	• Frailty Index (30 variables)	• FI mean ± SD: 0.54 ± 0.13.	• There were no significant differences in the frailty index among those with RRMS, PPMS, and SPMS *(p* = 0.948). • There was no observed relationship between frailty and disease duration *(p* = 0.706). • A significant association was found between frailty index scores and the number of falls experienced in the preceding six months *(p* = 0.030). • Participants were found to meet objective diagnostic criteria for severe (91.1 %) and moderate (8.9 %) frailty. • No correlation was found between the frailty index and the duration of using the mobility aid *(p* = 0.584).
Hanlon et al., 2018	To investigate the relationship between frailty, multimorbidity, specific long-term conditions, and mortality in middle-aged and older adults.	Physical frailty	Modified Fried frailty phenotype.	• Frailty prevalence: 17.0 % (in pwMS)	Frailty prevalence was higher in long- term conditions such as multiple sclerosis. • The top five long-term conditions associated with frailty were multiple sclerosis, chronic fatigue syndrome, chronic obstructive pulmonary disease, connective tissue disease, and diabetes. PwMS had the highest prevalence (17 %).
Zanotto et al., 2022a, b	To investigate the relationship between frailty and a history of falls among people living with multiple sclerosis.	Deficit accumulation model	• Frailty Index (40 variables)	• FI mean ± SD: 0.32 ± 0.14 Frailty prevalence: 66.1 %	• 56.9 % of participants reported at least one fall within the last 12 months, with a higher proportion of participants having severe frailty (73.8 %). • A moderate correlation between the frailty index and the EDSS was found (*p* < 0.001). • Following adjustments for age, sex, and EDSS, the frailty index was significantly associated with higher falls (*p* < 0.001).
Zanotto et al., 2023	To explore the association between frailty and the quantity and quality of free-living walking and the mediating influence of frailty on the relationship between disability and walking performance in people with multiple sclerosis.	Deficit accumulation model	Frailty Index (38 variables)	• FI mean ± SD: 0.32 + 0.14	• Participants with moderate and severe frailty demonstrated lower performance across all measures of walking quantity and quality (*p* < 0.001), with the exception of sample entropy *(p* = 0.187), compared to non-frail participants. • Frailty did not mediate the relationship between disability (EDSS) and measures of walking quality. • Frailty mediated the relationship between disability and measures of walking quantity, such as daily step counts and signal vector magnitude.
Kumar et al., 2024	To explore the relationship between frailty and sleep quality in obese people with MS.	Deficit accumulation model	• Frailty Index (41 variables)	• FI mean ± SD: 0.24 ± 0.11 Frailty prevalence: 32.5 %	• The FI and global PSQI scores were significantly associated (*p* < 0.05); the higher the FI scores, the higher the PSQI scores (i.e., lower sleep quality). • Frail pwMS had lower subjective sleep quality, higher sleep latency, lower habitual sleep efficiency, more sleep disturbances, higher use of sleep medications, and greater daytime dysfunction (*p* < 0.05). • Participants with frailty had a higher BMI than those without frailty (*p* < 0.001).
Palladino et al., 2022	To evaluate whether there is an additive or synergistic association between depression, frailty, vascular disease, and mortality in pwMS.	Deficit accumulation model	electronic Frailty Index (Clegg et al., 2016)	• Frailty prevalence: 3.8 %	• At the time of diagnosis, 3.8 % of the participants were frail, and 1.0 % had frailty and depression. • Frailty was associated with more primary care visits. • Frail pwMS with or without depression had a higher risk of all-cause mortality. • Frailty combined with depression significantly increased mortality risk, particularly in women.

*Notes:* FP: Fried Frailty phenotype, FI: Frailty Index, EDSS: Expanded Disability Status Scale, BMI: body mass index, PSQI: Pittsburgh Sleep Quality Index questionnaire, PDDS: Patient Determined Disease Step.

## Appendix A

**Table:**

Database	Syntax	No. of articles
PubMed	((multiple sclerosis [mh] OR “multiple sclerosis”[Title/Abstract] OR “MS”[Title/Abstract] OR “demyelinating disease”[Title/Abstract] OR “autoimmune neurological disorder”[Title/Abstract] OR “central nervous system demyelination”[Title/Abstract] OR “neuroinflammatory disease”[Title/Abstract])) AND (Frailty[mh] OR Frail Elderly[mh] OR Frail*[All] OR sarcopenia[mh] OR Sarcop*[All])	1618
Scopus	TITLE-ABS(“multiple sclerosis”) AND (ALL(sarcop*) OR ALL(Frail*) OR ALL(Debilit*))	1796
Web of Science	#1 (TS=(“MS”)) OR TS=(“multiple sclerosis”) #2 (ALL=(Frail*)) OR ALL=(Sarcop*) #1 AND #2	1473
PEDro	“multiple sclerosis” AND frailty	8
Embase	’multiple sclerosis’ AND (sarcop* OR frail* OR debilit*)	1436
Ovid MEDLINE	((multiple sclerosis or “multiple sclerosis” or “MS” or “demyelinating disease” or “autoimmune neurological disorder” or “central nervous system demyelination” or “neuroinflammatory disease”)).ab. AND ((Frailty or Frail Elderly or Frail* or sarcopenia or Sarcop*)).af.	1076
CINAHL	AB ((multiple sclerosis OR “multiple sclerosis” OR “MS” OR “demyelinating disease OR ”autoimmune neurological disorder“ OR ”central nervous system demyelination” OR “neuroinflammatory disease”))) AND TX ((Frailty OR Frail Elderly OR Frail* OR sarcopenia OR Sarcop*))	219
CENTRAL	((“Multiple sclerosis” OR “MS” OR “demyelinating disease” OR “autoimmune neurological disorder” OR “central nervous system demyelination” OR “neuroinflammatory disease”)):ti,ab,kw AND ((Frail* OR Debilit* OR sarco*))	343
clinicaltrials.gov	(“Multiple sclerosis” OR “MS” OR “demyelinating disease” OR “autoimmune neurological disorder” OR “central nervous system demyelination” OR “neuroinflammatory disease”) AND (Frail* OR Debilit* OR Sarco*)	30
